# Rare ovarian lesion in an adolescent girl

**DOI:** 10.4103/0971-9261.43031

**Published:** 2008

**Authors:** Senthilnathan Ramasamy, Jeyakumar Paramasivam, Krishnamohan Janardhanam

**Affiliations:** Department of Pediatric Surgery, Madras Medical College, Institute of Child Health and Hospital for Children, Egmore, Chennai - 600 008, Tamil Nadu, India

**Keywords:** Malignancy, ovarian edema, torsion

## Abstract

Large solid ovarian lesions are considered malignant in nature in pediatric and adolescent age group. We present an adolescent girl who had large solid ovarian lesion, with negative tumor markers. She underwent laparotomy and right oopherectomy. Histopathology revealed that the lesion was massive ovarian edema. This is an extremely rare lesion of ovary and is benign in nature. Very few case reports are available in English literature. Hence we suggest that massive ovarian edema should be considred as one of the differential diagnosis in all the patients having large solid ovarian lesions with ngative tumor marker assay. Ovarian preservation with the help of frozen section analysis should always be considred in these patients.

## INTRODUCTION

Massive ovarian edema[1] (MOE), refers to tumor-like enlargement of one ovary, or occasionally both, due to accumulation of edema fluid in the stroma. Although, there is still uncertainty about its pathogenesis, intermittent torsion of the ovary resulting in interference with venous and lymphatic drainage is a likely explanation. We report the case of an adolescent girl with unilateral massive ovarian edema along with a short discussion on the probable aetiopathogenesis. The rarity of the lesion has prompted us to report this case.

## CASE HISTORY

A 15-year-old girl presented to our outpatient department with complaints of lower abdominal pain and fullness of lower abdomen of one-month duration. Abdominal pain was constant, dull aching in nature with occasional exacerbation and with no radiation to the other areas. The lower abdominal fullness persisted, even after voiding. She had attained menarche, a year ago and her periods were moderate, regular and painless. On general examination her habbitus was normal. Abdominal examination revealed a solid mass in lower abdomen. The lower limit of the mass was not felt by palpation. External genitalia on inspection were found to be normal. On rectal examination, the mass was felt anteriorly. Based on the large size of the lesion, which is a feature of malignant ovarian germ cell tumors in the pediatric and adolescent age group, investigations were aimed in that direction. Tumor markers that are characteristic of pediatric and adolescent germ cell tumors were done. Serum alpha -feto protein and serum beta-Hcg levels were within normal limits. Imaging studies like ultra sonogram revealed a uniform echo texture mass of size 15-20 cm. Left ovary and uterus were normal. Chest skiagram was normal and the patient was prepared for surgery.

Laparotomy on the patient revealed a right ovarian mass of size 15-20 cm having a smooth surface and not adherent to surrounding structures. It was resected easily along with right fallopian tube. Left ovary and uterus were normal and were preserved. Minimal free fluid was present and was sent for cytological examination.

**Figure 1:**
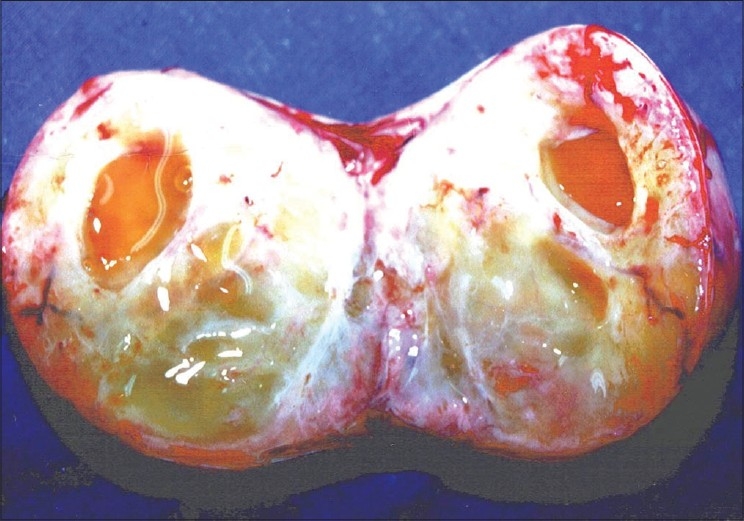
Cut section of the ovary showing small cystic spaces below the capsule and no hemorrhage was found. The stroma was fleshy

Cut section of the ovary showed small cystic spaces below the capsule and no hemorrhage was found. The stroma was fleshy [Figure 1]. The specimen was sent for histopathological examination. The postoperative period was uneventful and the patient recovered well. The patient was reviewed periodically, and at 5 years follow-up had no features of recurrence.

**Figure 2:**
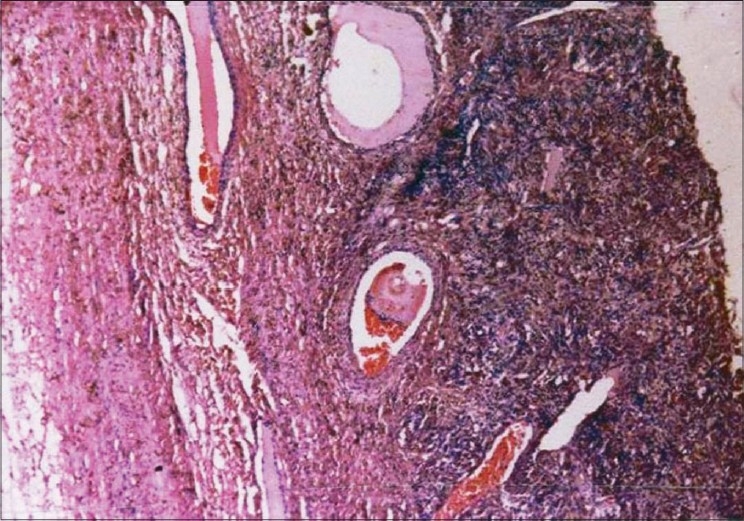
Histopathology. The pseudo capsule was composed of ovarian stroma, overlying markedly edematous ovarian stromal tissue with prominent vascular proliferation. This is a characteristic feature of stromal proliferation type of ovarian edema, (H&E)

The histopathological report was a surprise and the report was ovarian edema. The pseudo capsule was composed of ovarian stroma, overlying markedly edematous ovarian stromal tissue with prominent vascular proliferation. This is a characteristic feature of stromal proliferation type of ovarian edema [Figure 2]. Cytology report was negative for malignant cells.

## DISCUSSION

Massive ovarian edema is an extremely rare lesion of ovary. The most common age group of involvement is between 6 to 33 years with a median age of 20 years. There have been two types of presentation. The more common presentation has been acute or chronic abdominal pain resulting from torsion of involved ovary. The second type of presentation is due to menstrual disturbances in the form of mennorhagia. Endocrine disturbances like virilization and sexual precocity can occur in pediatric patients. Our patient had one-month history of abdominal pain with occasional exacerbations, probably due to intermittent torsion of the involved ovary.

Two explanations have been offered for Massive ovarian edema.[2] Intermittent torsion with a lymphatic obstruction causes a normal ovary to become edematous and the presence of lymphatic fluid stimulates stromal proliferation and occasionally focal luteinization. The basic process is stromal hyperplasia or hyperthecosis and the edema of the ovary is a secondary phenomenon, possibly due to torsion of an abnormally enlarged organ. Those who favor the first explanation opine that the process may be unilateral even in the presence of endocrine manifestations, whereas stromal hyperplasia and hyperthecosis are bilateral. The votaries of second explanation reply that in unilateral occurrence, the opposite ovary's wedge biopsy if done, on microscopic examination may reveal stroma hyperplasia.

## CONCLUSION

Massive ovarian edema case reports are very few in the literature. It is important to recognize and bear in mind during work up of a patient whose ultra sonogram shows an ovarian mass of uniform echogenicity with negative tumor markers. In case of a strong suspicion of this lesion at laparotomy, one should proceed to frozen section. If the report confirms ovarian edema, then ovarian size reduction procedure must be done for moderate size lesion. A very large lesion like our case, may not be amenable to conservative procedure and ovary may have to be excised. Contra lateral oophoropexy is a controversial treatment for the remaining ovary.[3] Abundant precaution must be exercised in preserving the opposite ovary and uterus. Ultra sonogram examination at regular intervals is mandatory in the follow-up period because the opposite ovary may develop this lesion.
